# The Diagnostic Odyssey in Children and Adolescents With X-linked Hypophosphatemia: Population-Based, Case–Control Study

**DOI:** 10.1210/clinem/dgae069

**Published:** 2024-02-09

**Authors:** Freya Boardman-Pretty, Ashley Kieran Clift, Hadley Mahon, Nadine Sawoky, M Zulf Mughal

**Affiliations:** Mendelian, London, EC1V 9EY, UK; Mendelian, London, EC1V 9EY, UK; Mendelian, London, EC1V 9EY, UK; International Medical Affairs, Kyowa Kirin, Galashiels, TD1 1QH, UK; Department of Paediatric Endocrinology & Metabolic Bone Diseases, Royal Manchester Children’s Hospital, Manchester, M13 9WL, UK; The Faculty of Biology, Medicine & Health, University of Manchester, Manchester, M13 9PL, UK

**Keywords:** X-linked hypophosphatemia, electronic healthcare records, primary care

## Abstract

**Context:**

X-linked hypophosphatemia (XLH) is a rare genetic disorder causing renal phosphate wasting, which predicates musculoskeletal manifestations such as rickets. Diagnosis is often delayed.

**Objective:**

To explore the recording of clinical features, and the diagnostic odyssey of children and adolescents with XLH in primary care electronic healthcare records (EHRs) in the United Kingdom.

**Methods:**

Using the Optimum Patient Care Research Database, individuals aged 20 years or younger after January 1, 2000, at date of recorded XLH diagnosis were identified using Systematized Nomenclature of Medicine Clinical Terms (SNOMED)/Read codes and age-matched to 100 controls. Recording of XLH-related clinical features was summarized then compared between cases and controls using chi-squared or Fisher's exact test.

**Results:**

In total, 261 XLH cases were identified; 99 met the inclusion criteria. Of these, 84/99 had at least 1 XLH-related clinical feature recorded in their primary care EHR. Clinical codes for rickets, genu varum, and low phosphate were recorded prior to XLH diagnosis in under 20% of cases (median of 1, 1, and 3 years prior, respectively). Rickets, genu varum, low phosphate, nephrocalcinosis, and growth delay were significantly more likely to be recorded in cases.

**Conclusion:**

This characterization of the EHR phenotypes of children and adolescents with XLH may inform future case-finding approaches to expedite diagnosis in primary care.

X-linked hypophosphatemia (XLH) is a rare, multisystem, X-linked, dominantly inherited disorder characterized by chronic hypophosphatemia. The incidence of XLH has been estimated as between 1.5 and 4.8 per 100 000 persons (1-3). It is caused by inactivating pathogenic variants of the Phosphate Regulating Endopeptidase Homolog, X-Linked (*PHEX*) gene (4), which results in increased plasma levels and expression of fibroblast growth factor 23 (FGF23). Raised plasma FGF23 reduces renal phosphate reabsorption by downregulating sodium/phosphate cotransporters NPT2a and NPT2c in the proximal renal tubules, resulting in increased urinary phosphate excretion and hypophosphatemia (5). Raised plasma FGF23 also results in reduced plasma levels of 1,25-dihydroxy vitamin D, the active metabolite of vitamin D, which in turn leads to impaired intestinal phosphate absorption. Chronic hypophosphatemia resulting from increased renal phosphate wastage and reduced intestinal phosphate absorption results in failure of the mineralization of the growth plate and osteoid in a growing child, resulting in rickets (6). In older adolescents and adults, XLH results in chronic osteomalacia (6).

Children with XLH develop genu varum or bow leggedness upon weight-bearing, abnormal head shape due to craniosynostosis, progressive and disproportionate decline in linear growth, muscle weakness, and bone pain and dental abscesses due to impaired mineralization of enamel and dentine (6). Adolescents and adults with XLH develop chronic pain, fatigue, and impaired mobility. Adults may experience debilitating sequelae of XLH, including hearing loss, tinnitus, vertigo, as well as symptoms of osteoarthritis, enthesopathies, Chiari type 1 malformations, and associated syringomyelia and spinal stenosis (6).

Despite the pathogenic variants in the *PHEX* gene being completely penetrant, genotype–phenotype correlations are complex and XLH can vary in its floridity of presentation (6, 7). This, in conjunction with the rarity of the condition, may predicate diagnostic delay in young people, and this period of diagnostic delay may be referred to as a “diagnostic odyssey.” Delayed diagnosis not only impacts the severity of progressive defects and quality of life (8), but is crucial to consider in the context of the recently approved novel therapeutic agent burosumab, a monoclonal IgG1 antibody that neutralizes FGF23 (9, 10).

Large-scale population-level primary care electronic healthcare record (EHR) datasets offer opportunities to characterize the epidemiology, clinical trajectories, and diagnostic odyssey of individuals with rare diseases (11). The scale of coverage permits the collation of case series larger than those which might be attainable with other approaches, and the source of data collection aligns with the setting in which much of the prediagnostic patient journey occurs, or where population-scale case-finding tools could be deployed. In this study, we use routinely collected, deidentified primary care data from the Optimum Patient Care Research Database (OPCRD), which covers over 22 million individuals in the United Kingdom (12), to characterize the EHR phenotypes and diagnostic odyssey for children and adolescents with XLH.

## Materials and Methods

The objectives of this study were to (1) identify a cohort of children and adolescents with a diagnosis of XLH recorded in their primary care record; (2) describe the recording of XLH-related clinical events in primary care for this XLH cohort; and (3) compare the recording of XLH-related clinical features in cases and healthy age-matched controls.

### Data Source

This study used the Optimum Patient Care Research Database (OPCRD), which collates deidentified, routinely collected primary care data from over 1100 general practices in the United Kingdom (13). There is coverage across all 4 UK nations and all leading general practice software systems (ie, EMIS, TPP, SystmOne). The database collects information on demographics, referrals, prescriptions, measurements, and clinical events using health care practitioner–entered clinical codes (ie, from the Systematized Nomenclature of Medicine Clinical Terms [“SNOMED”] and CTV3 [“Read”] coding systems). These are both terminologies comprising a “dictionary” of codes that can be used to encode clinical events in EHRs. Read codes were in widespread use in UK primary care until 2018, after which most coding was switched to use SNOMED system. OPCRD is broadly representative of the UK population in terms of age, sex, ethnicity, and socioeconomic status, and the clinical data it stores are representative of routine primary care, thus enabling studies of health care utilization patterns.

### Case Identification and Clinical Feature Definitions

Individuals with XLH were defined as those with the codes listed in Table 1 in their EHR. The date of the earliest recorded diagnostic code was used.

**Table 1.** SNOMED and read (CTV3) codes used to define cases of X-linked hypophosphatemia in the study

Individual	Individual	Individual
SNOMED	82236004	Familial X-linked hypophosphatemic vitamin D refractory rickets
SNOMED	855451000000106	X-linked hypophosphatemic rickets
CTV3	X40Qq	X-linked hypophosphatemic rickets

Abbreviation: SNOMED, Systematised Nomenclature of Medicine Clinical Terms.

As patients in OPCRD are deidentified at practice level and may change primary care provider over time, potentially duplicate records (ie, from the same individual being registered at multiple practices) were considered. XLH cases with the same year of birth (the granularity provided by OPCRD for purposes of anonymity) and same date of diagnosis were compared to calculate the total pairwise overlap of clinical events in their EHRs. Those with 10% overlap or more were merged into a single case. To calculate age at diagnosis/age at occurrence of clinical features, patients were assigned a date of July 2 (midpoint of year) to their year of birth.

To analyze data within a single coding system, all clinical event records with a CTV3 code present were mapped to their corresponding SNOMED codes using reference tables generated from NHS Digital's TRUD reference data.

**Figure 1.**
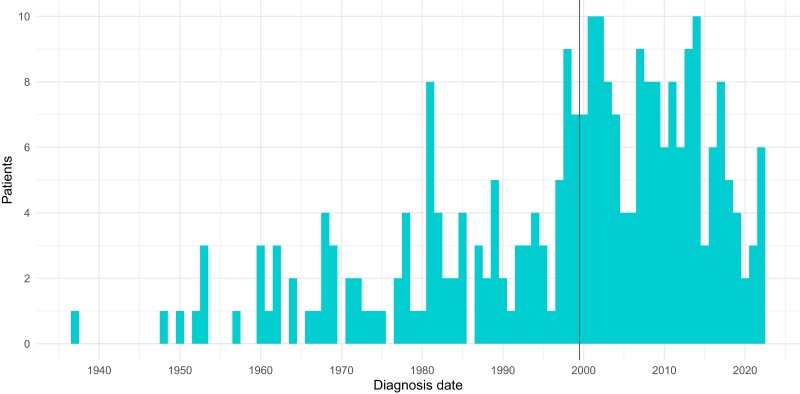
Distribution of year of diagnosis for the 261 cases of X-linked hypophosphatemia as recorded in the Optimum patient care database. The vertical black line refers to the eligibility date for inclusion in the final cohort (cases diagnosed on January 1, 2000 or later).

Due to considerations of data quality and improvements in clinical coding over time by general practitioners (Figure 1 (13)), the deduplicated cohort was filtered to a final cohort comprising those diagnosed on or after January 1 2000, and aged under 20 years at diagnosis.

### Clinical Feature Definitions

XLH-related clinical features of interest were compiled following literature review (including review of Human Phenotype Ontology, https://hpo.jax.org/app/) and consultation with M.Z.M. These comprised arthritis, craniosynostosis, delayed ability to stand, disproportionate short stature/growth delay, enthesitis, flattening of the talar dome, fractures, the lower limb deformities genu valgum and genu varum, insufficiency fractures, joint or bone pain, low phosphate, nephrocalcinosis, periodontal disease, rickets, sensorineural hearing impairment, and tooth problems. These were defined as the presence of relevant SNOMED codes in EHRs (see code lists in Table 1 (13)), apart from hypophosphatemia, increased bone mineral density, and disproportionate short stature/growth delay.

Hypophosphatemia was defined as the presence of a relevant SNOMED code, and/or a recorded serum phosphate level below age-specific reference ranges (Table 2 (13)). Short stature/growth delay was defined as presence of relevant SNOMED codes, or a measured height below the 25th percentile. Bone mineral density was based on recorded results from dual-energy x-ray absorptiometry scans (Z score > 2).

### Case–Control Matching

In order to compare the frequency and temporality of XLH-related clinical events in cases to the remaining population, each XLH case was matched to 100 controls with the same year of birth, randomly selected from the OPCRD database. XLH cases had their diagnosis date as an index date for ascertaining prior clinical events. As controls were “never cases,” they could have contributed clinical event data throughout the study period up until age 20, whereas cases had their relevant EHR data truncated at their date of diagnosis. To account for these differences in time frames for clinical event recording, controls were assigned an index date drawn from a uniform distribution between the date of their first recorded clinical event and the latest of their practice leaving/date of last clinical event.

### Statistical Analyses

In the XLH cases, we undertook descriptive analyses regarding the frequency of clinical features being recorded at any point in their EHRs, their frequency of recording prior to diagnosis, and the median time between first recording relative to XLH diagnosis date.

To compare feature recording between cases and controls prior to the index date, chi-squared tests were used (or Fisher's exact test if cells in the 2 × 2 table contained counts <5). A threshold for statistical significance of *P* < .05 was used.

Data extraction was performed using the SQL programming language; subsequent data processing and analyses were undertaken using R.

### Ethics Approval and Reporting

Ethics approval for the OPCRD databases has been obtained from the NHS Health Research Authority (REC reference 20/EM/0148). This descriptive study was undertaken as part of the protocol approved by the Anonymised Data Ethics & Protocol Transparency (ADEPT) committee to evaluate case-finding tools for rare diseases including XLH (reference: 1221). The study is reported in accordance with the Strengthening the Reporting of Observation Studies in Epidemiology (STROBE) guidelines (14).

## Results

### Study Population and XLH Cases

At the date of data extraction (June 1, 2023), there were a total of 22 588 610 million patient records in OPCRD. 289 XLH cases were initially identified, with 28 ascertained to be duplicate records, yielding 261 cases with a coded prevalence of XLH of 1.16 per 100 000 in OPCRD. Of these, 67% of XLH cases were of female sex, which is similar to the female preponderance reported elsewhere (1).

**Figure 2.**
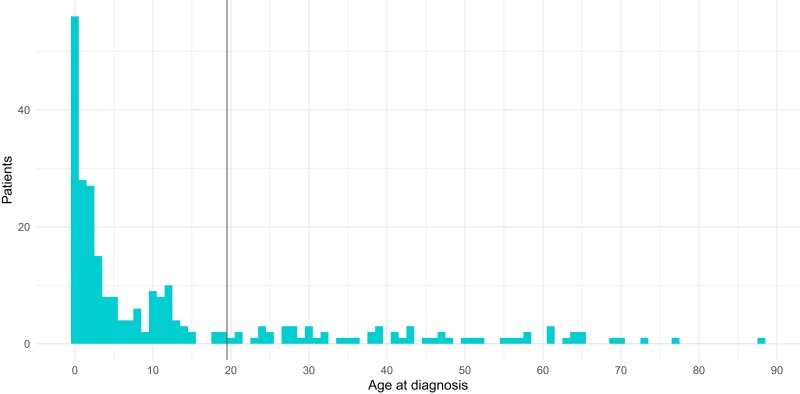
Age at first recorded diagnosis of X-linked hypophosphatemia in the Optimum Patient Care Research Database (OPCRD), as defined by presence of relevant SNOMED/CTV3 codes, as per Table 1. The vertical black line corresponds to cut-off used to define the final study cohort of interest, in other words, those aged 20 or lower at first recorded diagnosis, diagnosed after January 1, 2000. There were 261 cases of XLH recorded in OPCRD overall, out of a total of 22.59 million individuals.

Recorded XLH diagnosis dates ranged between 1937 and 2022 (Fig. 1), while age at first record of the diagnosis ranged between 0 and 88, with a median age of 4 years (Fig. 2). After filtering the cases to those aged <20 at diagnosis and diagnosed after January 1, 2000, following consideration of data quality (Figure 1 (13)), 99 XLH cases were included in the final analyses.

### Recorded Clinical Features in the XLH Cohort

**Figure 3.**
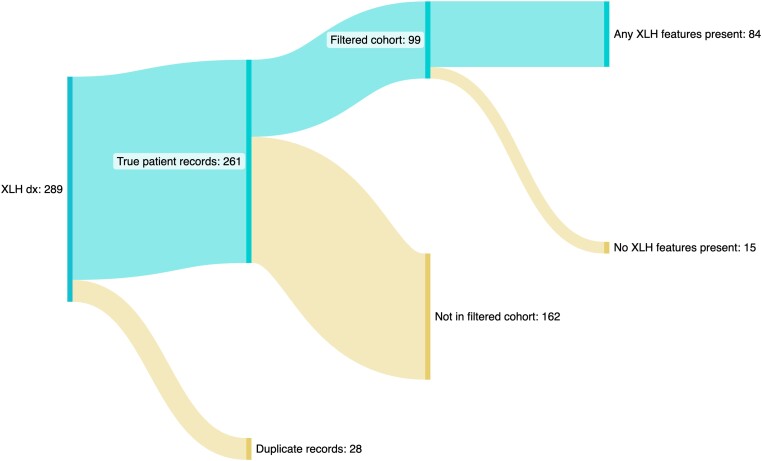
Sankey diagram summarizing the flow from initial record identification to handling of records deemed to be duplicate individuals, to final analysis cohort filtering, and recording of any of the 17 features of interest in the final study cohort.

Of the 99 XLH cases, 84 (84.85%) had at least 1 of the 17 XLH-related clinical features of interest recorded at any point in their primary care EHR (Fig. 3). Therefore, 15 of the XLH cases did not have any of these recorded at any point. Five features of interest were not recorded in the HER of any case, namely arthritis, flattening of the talar dome, increased bone mineral density, insufficiency fractures, and periodontal disease. Three of the 15 XLH cases without any XLH-related features coded in their primary care record had very sparse EHRs, with no significant coded medical history prior to the date of their recorded XLH diagnosis.

**Figure 4.**
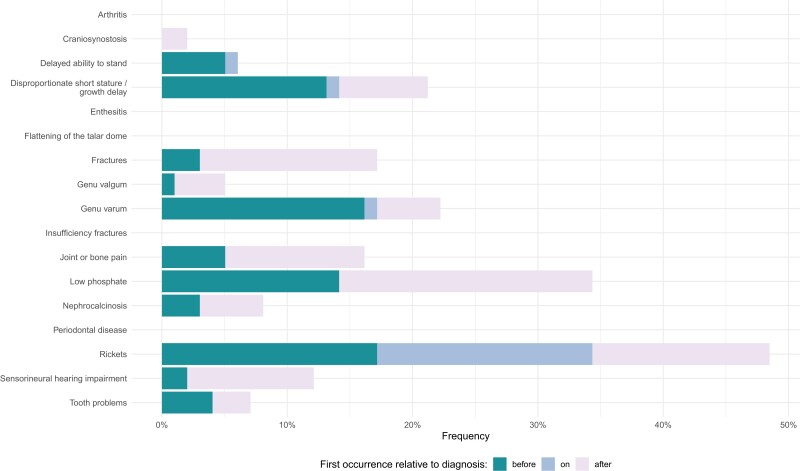
Frequency of clinical event recording in the final cohort of children and adolescents with X-linked hypophosphatemia, split by prediagnosis, at same date of diagnosis, and after diagnosis. “Arthritis” refers to degenerative osteoarthritis.

**Figure 5.**
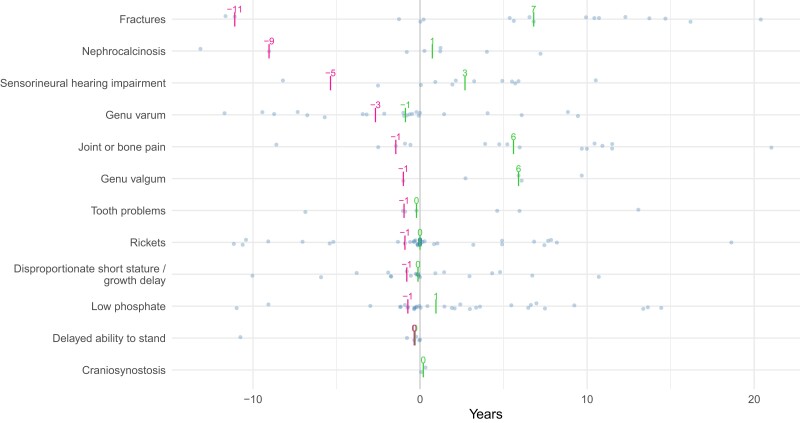
Time between recording of clinical features and recorded diagnosis of X-linked hypophosphatemia (XLH) in years. Red lines refer to median time prior to diagnosis that the clinical feature was recorded in the primary care electronic health care record (the denominators are the individuals that had the feature coded prior to diagnosis), and the adjacent numbers represent median years prior to diagnosis. The green dashes and adjacent numbers refer to the median years relative to the diagnosis that the clinical features were recorded without a time restriction being applied. Here, for the green dashes and numbers, the denominators were those that ever had the feature recorded, either prior to or after the XLH diagnosis date.

Figure 4 shows the frequencies of clinical feature recording relative to XLH diagnosis (before, on date thereof, and after). Rickets, low phosphate, short stature, and genu varum were the features most commonly recorded at any point in XLH cases’ EHRs. Of note, 36% of XLH patients had rickets first coded on the same day as their XLH diagnosis. If coded, craniosynostosis was only recorded after XLH diagnosis. Fractures, joint or bone pain, low phosphate, and sensorineural hearing impairment were more commonly recorded after XLH diagnosis. Interestingly, under 48% of XLH cases had rickets coded in their EHR. Most features were coded a median of 1 year prior to XLH recording (eg, joint or bone pain, tooth problems, rickets, short stature, and low phosphate) (Fig. 5). For many features, particularly rickets, low phosphate, short stature, and genu varum, there appeared to be some clustering of first appearances of relevant codes around the year prior to XLH diagnosis.

It is expected that for some patients with XLH, a clinical diagnosis may lead to genetic testing for confirmatory genotyping. As no SNOMED codes exist specifically regarding genetic diagnosis of XLH, we explored coded referrals and other EHR features that may indicate engagement with clinical genetics teams. Nine of the 99 XLH cases had such events coded in their EHRs, 4 of which were within 2 years after the initial diagnosis code. Due to small counts in the context of avoiding potential identification, we do not present more granular data here on timing of genetic referrals for these cases.

Regarding orthopedic surgical intervention for lower limb deformities, we were unable to explore which children underwent specific surgeries at any stage in the disease, due to data availability. We were however able to identify that 50 individuals of the final XLH group had codes for orthopedic service referrals recorded in their primary care notes at any point, with 14 of these individuals being referred prior to their XLH diagnosis date.

### Recording of XLH-Related Features in Cases and Controls

**Figure 6.**
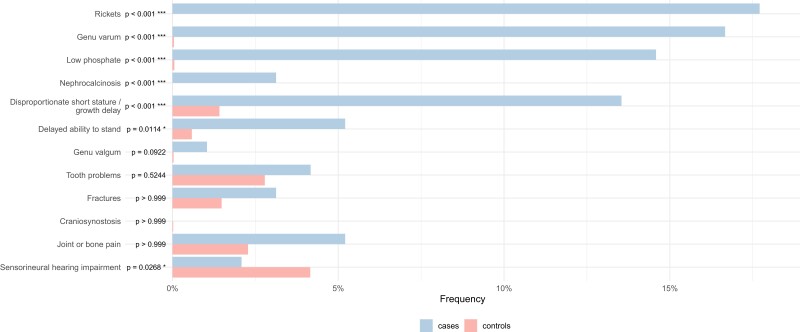
Comparisons of the frequencies of clinical feature coding prior to the index date between cases and controls. For the X-linked hypophosphatemia cases, the index date was the first recorded instance of a relevant clinical code. For the controls, the index date was a randomly assigned date drawn from a uniform distribution between the first eligible clinical entry and last clinical entry in the primary care record.

Figure 6 summarizes the results when comparing prevalence of XLH-related clinical features in cases and controls prior to the index date—rickets, genu varum, low phosphate, nephrocalcinosis, short stature, and delayed ability to stand were all significantly more likely to be recorded in XLH cases than in controls (all *P* < .05). There were no significant differences observed between preindex date recording of tooth problems (*P* = .52), fractures (*P* = .99), craniosynostosis (*P* = .99), and joint or bone pain (*P* = .99) between cases and controls, and sensorineural hearing impairment was more likely to be recorded in controls than cases (*P* = .03). The latter likely partly reflects other causes of sensorineural hearing loss being more common in the wider pediatric population than XLH-associated hearing loss in conjunction with primary care recording behaviors.

## Discussion

Using a population-level primary EHR database, this study sought to characterize the diagnostic odyssey of children and adolescents diagnosed with XLH in the United Kingdom since 2000. This included an exploration of the temporality of coding of clinical features of the condition, and a comparison of clinical feature recording frequency between cases and controls in order to understand the scope for clinical decision support (“case-finding”) tools that could support expedited diagnosis in the future. Key findings of this study include observing that some archetypal XLH features such as rickets are only recorded in primary care EHR data prior to XLH diagnosis in under 20% of individuals, but, despite this, several features might be discriminatory between cases and controls, with potential to inform approaches to case-finding algorithms.

The coding-defined prevalence of XLH in this study (1.16 per 100 000) is similar but slightly lower than some previous estimates from national epidemiological studies. One such study from Norway used an ICD-10 code (E83.3—disorders of phosphorus metabolism and phosphatases) combined with relevant exclusions such as hypophosphatasia, and identified 21 cases and estimated a prevalence of 16.6 per million (1.66 per 100 000) (3). The prevalence from the present study is similar to another study by Hawley and colleagues that also used primary care data (Clinical Practice Research Datalink, CPRD GOLD) in the United Kingdom (1). This reported a childhood prevalence of 15.1 per million (95% CI: 11.3 to 20.1; 1.51 per 100 000) in the years 2012-2016. In contrast to the present study's approach of using curated SNOMED and Read codes, the study of Hawley and colleagues used an algorithmic approach to ascertain potential XLH cases from the CPRD based on Read codes including active rickets, osteomalacia, vitamin-D-resistant rickets, and laboratory test values (eg, phosphate) (1). Based on this, of 522 initially identified potential cases, 122 were used in their final analyses, classified into “highly likely” (n = 27), “likely” (n = 37), and “possible” (n = 58) XLH cases. This study was primarily focused on premature mortality in XLH cases, and while it did report on “ever recording” of features such as hypercalciuria and use of vitamin D, the present study was focused on deeper aspects of the EHR phenotypes of XLH cases.

The strengths of this study include the size, coverage, and nature of the database used. The use of a dataset containing over 22 million patient records permitted the collation of an XLH cohort of 99 children and adolescents in the final analyses of this rare disease. OPCRD collects routinely collected data from general practices across the United Kingdom, thereby minimizing selection, recall, and respondent biases. Further, a preferable deployment setting for any large-scale rare disease case-finding tool is in primary care, given that this is where the majority of clinical contacts occur in the NHS, where diagnosis might be delayed, and where consultations for potential disease-related symptoms or signs are most commonly initiated.

The use of routinely collected coded, primary care data also presents some important limitations worthy of consideration. First, this study was not able to access free clinical text in primary care notes—the reliance on clinical coding by individual health care practitioners may mean that there is some misclassification bias for the clinical features explored, for instance, rickets may have been noted in free text, but not had a SNOMED/Read code inputted. Alternatively, due to the incompletely specific nature of the SNOMED codes used to define XLH, there may be some individuals with rarer, autosomal forms of hypophosphatemic rickets (6) included in the final XLH cohort. As XLH is the commonest form of familial/genetic hypophosphatemia, we expect the vast majority of XLH cases here to be true cases. Second, at the time of data extraction and study conduct, this dataset was not able to be linked to secondary care datasets such as NHS Digital's Hospital Episodes Statistics. This would have likely increased the ascertainment of XLH cases by identifying those diagnosed in secondary/tertiary care but not coded in the general practitioner’s record. Third, primary care EHR studies typically make the implicit assumption that the first date of code recording represents the appearance of the feature of interest. Fourth, we did not link the age of diagnosis to the family history of XLH; this may be important as delayed diagnosis is more common in patients with de novo *PHEX* mutations. Overall, reliance on coded primary care data may be associated with inaccuracies and biases inherent to health care practitioners manually imputing codes—there may be inter individual and intraindividual variation between practitioner's proclivities to code a given clinical event (eg, thresholds, unconscious bias, “coding style”), variation in what kinds of clinical events are coded (eg, some practitioners may mostly code diagnoses, rather than symptoms), or incorrect codes being selected. Such biases in clinical coding may, in general, affect estimation of parameters such as prevalence of symptoms or regression model coefficients, depending on the study aims. In the present study, our intentions were to describe the extent of symptom recording in XLH in routinely collected primary care data “as is,” and explore whether these data could support the development of tools with clinical usefulness. There will be inaccuracies in the way that these data may be coded, but that does not preclude the data being of potential utility.

In conclusion, this study provides evidence regarding XLH clinical feature recording in routine primary care records—these data may support better understanding of the epidemiology of this rare condition or inform new approaches to early case-finding. The latter is of importance as early initiation of treatment in children with XLH is associated with better outcomes, including improved height and reduced skeletal deformities.

## Data Availability

Due to data access licensing and data security, raw study data cannot be made available to other investigators. Regulations regarding access to and use of the Optimum Patient Care Research Database are available here: https://www.opcrd.optimumpatientcare.org/licenses

## Abbreviations

FGF23: fibroblast growth factor 23
EHR: electronic healthcare record
OPCRD: Optimum Patient Care Research Database
SNOMED: Systematized Nomenclature of Medicine Clinical Terms
XLH: X-linked hypophosphatemia
